# Mitigating Wireless Channel Impairments in Seismic Data Transmission Using Deep Neural Networks

**DOI:** 10.3390/s21186105

**Published:** 2021-09-12

**Authors:** Naveed Iqbal, Abdulmajid Lawal, Azzedine Zerguine

**Affiliations:** 1 Department of Electrical Engineering, King Fahd University of Petroleum & Minerals, Dhahran 31261, Saudi Arabia; g201403800@kfupm.edu.sa (A.L.); azzedine@kfupm.edu.sa (A.Z.); 2 Center for Energy and Geo Processing, King Fahd University of Petroleum & Minerals, Dhahran 312161, Saudi Arabia; 3 Center for Communication Systems and Sensing, King Fahd University of Petroleum & Minerals, Dhahran 31261, Saudi Arabia

**Keywords:** deep neural networks, wireless geophones, channel noise

## Abstract

The traditional cable-based geophone network is an inefficient way of seismic data transmission owing to the related cost and weight. The future of oil and gas exploration technology demands large-scale seismic acquisition, versatility, flexibility, scalability, and automation. On the one hand, a typical seismic survey can pile up a massive amount of raw seismic data per day. On the other hand, the need for wireless seismic data transmission remains immense. Moving from pre-wired to wireless geophones faces major challenges given the enormous amount of data that needs to be transmitted from geophones to the on-site data collection center. The most important factor that has been ignored in the previous studies for the realization of wireless seismic data transmission is wireless channel effects. While transmitting the seismic data wirelessly, impairments like interference, multi-path fading, and channel noise need to be considered. Therefore, in this work, a novel amalgamation of blind channel identification and deep neural networks is proposed. As a geophone already is responsible for transmitting a tremendous amount of data under tight timing constraints, the proposed setup eschews sending any additional training signals for the purpose of mitigating the channel effects. Note that the deep neural network is trained only on synthetic seismic data without the need to use real data in the training process. Experiments show that the proposed method gives promising results when applied to the real/field data set.

## 1. Introduction

Classical seismic acquisition networks rely on cable-based systems. With the increase in the surveying area, cable-based systems are not practical owing to the weight and cost of the cables. Therefore, there is a need for a robust wireless seismic network that can transmit hundreds of recordings from geophones to the data center. This poses two major challenges: the data from a geophone must reach the data center in a timely manner while retaining the quality. The timing issue has been addressed in the previous studies [1,2]. However, the wireless transmission also requires mitigating the channel effects, like interference, multi-path fading, and noise. In traditional wireless systems, periodic training signals are transmitted in order to estimate and ultimately remove the channel effects. The inclusion of additional training signals on top of a giant amount of seismic data makes the situation even worse, i.e., more bandwidth and time are required for transmission [3]. None of the previous studies (see in [1,2], and references therein) focuses on this important issue. Most of the work done is related to the design of the wireless seismic system while completely ignoring the wireless link (channel) impairments.

In this work, efficient mitigation of the channel effects without using additional training signals is proposed. The proposed method relies on the blind system identification and deep neural networks approach. Blind system identification is a mature field [4] that estimates the channel without using any training signals. However, channel estimation using this method highly depends on the additive noise. Therefore, deep neural networks are used to alleviate this vulnerability.

Deep neural networks require data for training in a supervised learning setup [5,6]. In the proposed setting, synthetic data (generated using a realistic model) are used for this purpose. Therefore, once the network is trained offline, then it is suitable for real-time implementation without the need of field (real) data for training. The method consists of two deep neural networks: one is used for signal-to-noise ratio (SNR) enhancement and the other for classification. The blind system identification methods are effective in low-noise environments without the need for SNR improvement. As the SNR is not known at the data center, the classifier network decides whether to pass geophone data through the SNR enhancement network or not.

Furthermore, the geophone setup environment is stationary, i.e., geophones or the data center are fixed at the locations for several shots. This added advantage of a stationary environment is used together with blind system identification for improving the estimation of the channel impulse response.

The main contributions of this work are summarized as follows:

- Estimating the channel impulse response using blind identification method. The estimation is further improved by taking the stationary environment into account.
- Enhancing the SNR using deep convolutional neural networks by taking into account the features of seismic data in the frequency domain.
- Classification of geophone data using a deep fully connected neural network in order to decide about the need for SNR enhancement. This point addresses the practical implementation aspect of the proposed method.

The rest of the paper is organized as follows. The blind system identification is covered in Section 2. Section 3 discusses SNR enhancement, while Section 4 presents simulation results. Finally, Section 5 draws the conclusion.

## 2. Blind Channel Identification

Blind channel identification methods rely on a multichannel framework that is obtained either by using an array of antennas at the receiver side or oversampling the received signal. Both scenarios are best suited for the situation at hand. The reason being that the geophone already has to transmit a large amount of data under tight timing constraints [2] so oversampling at geophone is not feasible. Furthermore, oversampling or multiple antennas at a geophone also increases the processing load on battery-driven wireless geophone. Therefore, for multichannel blind system identification, the load (oversampling and multiple antennas) is shifted to the data center where power and processing requirements are relaxed.

Assuming that a single geophone data passes through *m* independent channels before reaching the data center (this is achieved by oversampling or array of antennas at the receiver). The discrete channel model for the window of *M* received samples is obtained by stacking the data into a vector/matrix representation and it is given as follows:

(1) $$\begin{array}{c} y_{M}\left(n\right)=H_{M}x_{M+L-1}\left(n\right)+z_{M}\left(n\right), \end{array}$$

where the received data is $y_{M}\left(n\right)={\left[y^{H}\left(n\right),\cdots ,y^{H}\left(n-M+1\right)\right]}^{H}$ and the transmitted digital modulated data is $x_{M+L-1}\left(n\right)={\left[x\left(n\right),\cdots ,x\left(n-M-L+2\right)\right]}^{T}$. The additive random noise $z_{M}\left(n\right)$ is stacked in a similar way to $y_{M}\left(n\right)$, and $H_{M}$ is an mM×(M+L−1) block Toeplitz matrix given as

(2) $$\begin{array}{c} H_{M}=\left[\begin{array}{ccccc} h(0) & \cdots & h(L-1) & \cdots & 0\\ \vdots & \ddots & \ddots & \ddots & \vdots \\ 0 & \cdots & h(0) & \cdots & h(L-1) \end{array}\right], \end{array}$$

where $y\left(n\right)={\left[y_{1}\left(n\right),\cdots ,y_{m}\left(n\right)\right]}^{T}$, $h\left(i\right)={\left[h_{1}\left(i\right),\cdots ,h_{m}\left(i\right)\right]}^{T}$, and $z\left(n\right)=[z_{1}\left(n\right)$, $\cdots ,z_{m}{\left(n\right)]}^{T}$. Let’s $h={\left[h^{T}\left(0\right)\cdots h^{T}\left(L-1\right)\right]}^{T}$ be the desired vector containing all the channel’s taps then the objective is to estimate these channels’ impulse responses, i.e., $\hat{h}$ using the observation data in (1). There are many subspace-based methods for blind system identification (see in [4] and references therein). Here, the method proposed in [4], namely, structured-based subspace method (SSS), is used. This method claims to be efficient in ill-conditioned channel matrices. In this approach, one searches for the system/channel matrix $H_{M}$ in the form $H_{M}=V_{x}Q$ such that the orthogonality criterion is set to be equal to zero, i.e., ${∥V_{z}^{H}{\hat{H}}_{M}∥}^{2}=0$. Furthermore, Q is chosen such that the resulting matrix is close to the desired block Toepliz structure. The columns of matrices $V_{x}$ and $V_{z}$ spans the signal and noise subspace, respectively.

Note that the geophones and the data center locations are fixed for several shots, therefore the channel impulse response is not expected to change. This additional advantage is used to further enhance the channel estimation $\hat{h}$. As the seismic shooting process is repeated over and over again, the channels’ impulse response for the $i^{th}$ shot is updated according to the following recursion

(3) $$\begin{array}{c} {\bar{h}}_{i}=\left(1-\frac{1}{\alpha }\right){\bar{h}}_{i-1}+\frac{1}{\alpha }{\hat{h}}_{i-1}, \end{array}$$

where ${\hat{h}}_{i}$ is the estimated channels’ impulse response using SSS method and ${\bar{h}}_{i}$ is the updated estimation at the $i^{th}$ shot. For seismic signal recovery, γ shots are used to get the final channel response ${\bar{h}}_{\gamma }$ and then optimum equalization method, i.e., maximum likelihood sequence estimation (MLSE) [7], is used to obtain a robust estimation of the transmitted signal $\hat{x}$. The MLSE is a computationally expensive technique; however, the equalization is done offline at the data center. Therefore, it is worth using the computationally intensive approach to achieve the best reconstruction of the seismic signal.

## 3. SNR Enhancement Using Deep Neural Networks

The aforementioned blind system identification together with the MLSE gives promising results under low noisy environments. However, the performance deteriorates under low SNR levels. Therefore, in this section, an SNR enhancement is introduced using deep neural networks. As the noise in (1) is random, the performance of the blind system identification method varies trace-by-trace (a trace is the acquired data by a geophone per shot). Traces that need SNR enhancement need to be differentiated from traces that do not. Therefore, traces are first classified based on the SNR level, and then the ones that need enhancement are fed to the SNR enhancement network. For this purpose, two neural networks are defined: one network classifies the traces and the other enhances the SNR. Raw seismic data are preprocessed before feeding to the deep neural networks.

### 3.1. Preprocessing Stage

For the SNR enhancement, a trace is transformed to the 2D domain. This is done using a short-time Fourier transform (STFT). However, using the Fourier transform requires using complex numbers that double the complexity. To overcome this issue, the discrete cosine transform is used instead. Therefore, it is called here as short-time discrete cosine transform (STDCT). STDCT is a sequence of discrete cosine transforms applied on windowed sections of the data and sliding the window through the entire record. Application of DCT on a windowed section of the data is called herein as a segment. The DCT type-IV [8] is defined for a window of size *N* as

(4) $$\hat{X}\left(k\right)=\sqrt{\frac{2}{N}}\sum_{n=1}^{N}\hat{x}\left(n\right)\cos\left(\frac{\pi }{4N}\left(2n-1\right)\left(2k-1\right)\right).$$

To find the inverse, *k* and *n* are switched in the above definition. The seismic waveforms are transformed to the time-frequency domain using the STDCT with a rectangular window of size N=128 samples and overlap of 90%. Using the STDCT, spectra of a trace in the time-frequency domain are obtained. The noisy spectra are used to get the clean spectra with the help of the deep neural network. To recover the time domain trace, a procedure similar to inverse STFT is performed [9].

For the classification, a 2048-point discrete Fourier transform (DFT) is performed on a whole trace and then deep neural network for classification decides about the further processing based on absolute value of the transform. DFT is performed as follows:

(5) $$\hat{C}\left(k\right)=\sum_{n=0}^{M-1}\hat{x}\left(n\right)e^{-j\frac{2\pi kn}{M}}k=0,1,\ldots ,2047,$$

where *M* is the trace length.

The reason for transformation to frequency domain is that the deep neural networks are able to better learn the specific frequency-domain seismic data characteristics/features and, thus, generalize well.

### 3.2. SNR Enhancement

Given segments of noisy spectra ${\left\{\hat{X}\left(n\right)\right\}}_{n=1}^{M/0.1N}$ and clean spectra ${\left\{X(n)\right\}}_{n=1}^{M/0.1N},$ our aim is to learn a mapping *f* which generates segments of ‘denoised’ spectra ${\left\{f\left(\hat{X}\left(n\right)\right)\right\}}_{n=1}^{M/0.1N}$ that approximate the clean spectra using the $\ell_{2}$ norm, e.g.,

(6) $$\begin{array}{c} \min\sum_{t=1}^{M/0.1N}{∥X\left(n\right)-f\left(\hat{X}\left(n\right)\right)∥}_{2}^{2}. \end{array}$$

Specifically, we formulate *f* using a deep neural network. If *f* is a recurrent type network, the temporal behavior of input spectra is already addressed by the network, and thus objective (6) suffices. On the other hand, for a convolutional type network, the past ⌊Δ/2⌋ noisy segments ${\left\{\hat{X}\left(i\right)\right\}}_{i=n-\lfloor \Delta /2\rfloor }^{n}$, the future ⌊Δ/2⌋ noisy segments ${\left\{\hat{X}\left(i\right)\right\}}_{i=n}^{n+\lfloor \Delta /2\rfloor }$ and the current noisy segment $\hat{X}\left(n\right)$ are considered to denoise the current segment of noisy spectra, e.g.,

(7) $$\begin{array}{c} \sum_{n=1}^{M/0.1N}{∥X\left(n\right)-f\left({\hat{X}}_{n-\lfloor \Delta /2\rfloor },\cdots ,{\hat{X}}_{n-\lfloor \Delta /2\rfloor }\right)∥}_{2}^{2}. \end{array}$$

**Figure 1:**
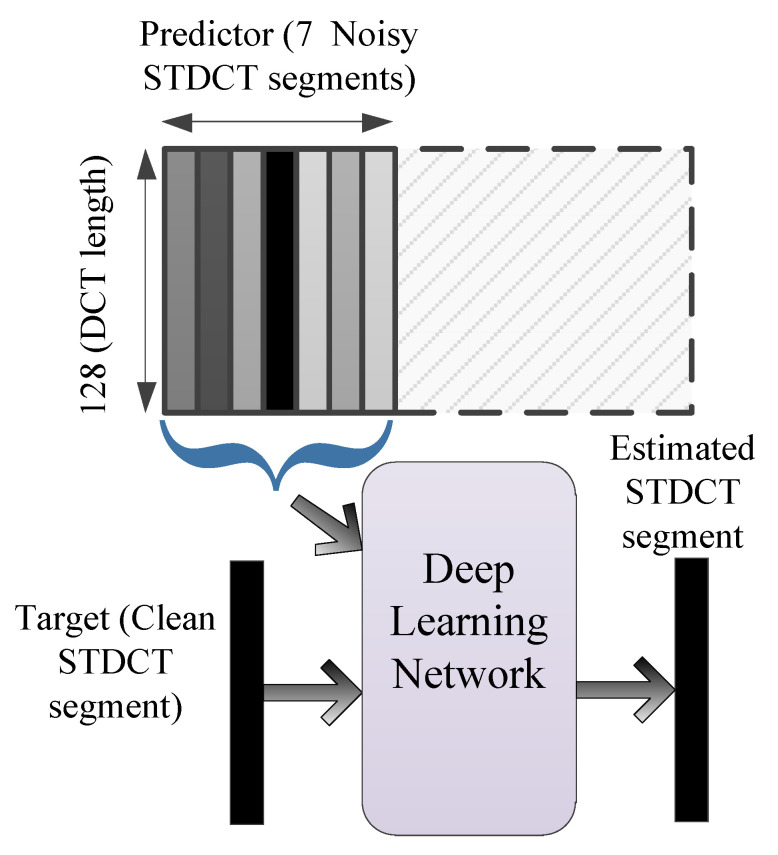
Schematic diagram of the denoising method. Seven STDCT segments are fed to the deep convolutional neural network to get a clean STDCT segment.

The value of Δ is set to 7, which is obtained empirically. The schematic diagram of the denoising network is depicted in Figure 1. The input to the network is 7 noisy STDCT segments and the output is the cleaned middle segment. The design of the denoising network in this way is similar to the filter design in digital signal processing [10] and makes more sense as compared to just having a network that maps input to the output for performing the denoising task.

**Figure 2:**
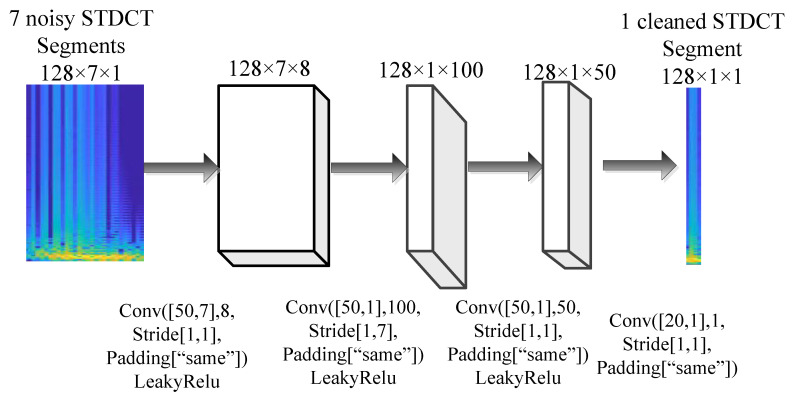
Deep convolutional neural network for SNR enhancement with convolutional (Conv) and LeakyRelu layers.

The fully convolutional network comprises 4 convolutional layers. The first 3 convolutional layers have a filter height of 50 each, and the number of filters is 8, 100, and 50, respectively. The last convolutional layer has a filter height of 20 with 1 filter. In this network, convolutions are performed in only one direction, i.e., along the frequency dimension, and the filter size along the time dimension is set to 1 (filter width = 1) for all layers except for the first layer it is 7. Each convolutional layer is followed by a leaky rectified linear unit (LeakyRelu) layer excluding the last one. The activation function LeakyRelu is defined as follows:

(8) $$g\left(s\right)=\left\{\begin{array}{cc} 0.01s & \mathrm{for}s<0\\ s & \mathrm{for}s\geq 0 \end{array}\right.$$

where *s* is input to the activation layer. The deep convolutional network is shown in Figure 2. Other parameters for the convolutional layers are as follows: stride is set to 1 along the horizontal and vertical directions for all layers except it is 7 along vertical direction for the second layer. Padding is added to get the output of the same size as the input when the stride equals 1. If the stride is larger than 1, then the output size is ⌈(inputSize/stride)⌉, where inputSize is the height or width of the input and stride is the stride in the corresponding dimension.

For optimization, the following parameters are used: convolutional layer weights are initialized as in [11] and biases are set to zeros. The network is trained using backpropagation with gradient descent optimization and adaptive learning rates, i.e., Adam algorithm [12] with a mini-batch size of 600. The initial learning rate is lr=0.001 with $\beta_{1}=0.9$, $\beta_{2}=0.999$, and $\epsilon =10^{-8}$. Furthermore, the learning rate is decreased by a factor of 0.9 every epoch. The training is stopped when validation loss does not decrease for more than 5 epochs. The loss function used to calculate the gradients is given as

(9) $$\begin{array}{c} E\left(\phi \right)=\sum_{q=1}^{128}\frac{1}{2}{\left[t\left(q\right)-p\left(q\right)\right]}^{2}. \end{array}$$

where *p* and *t* are the network prediction and target output, respectively. A regularization term is also added to the loss function E(ϕ) to reduce the overfitting problem [13], i.e., $E_{r}\left(\phi \right)=E\left(\phi \right)+\frac{\lambda }{2}ww^{T}.$ The regularization factor λ is set to 0.00005.

### 3.3. Classification

**Figure 3:**
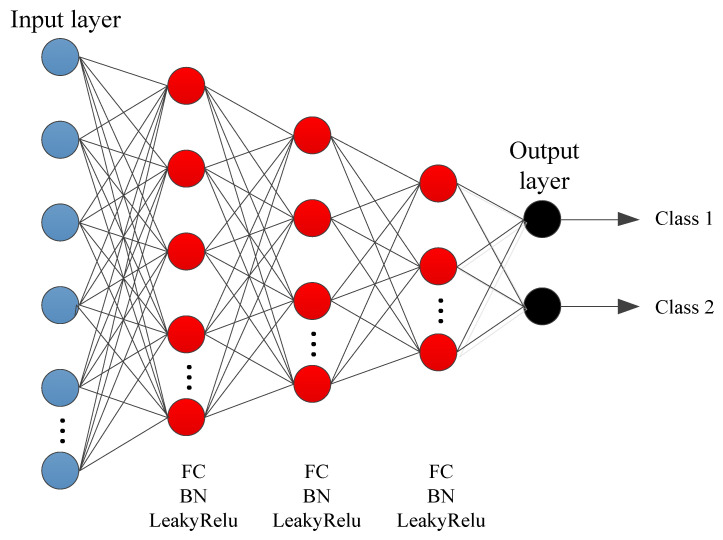
Fully connected neural network for classification.

The traces are classified based on the SNR after MLSE. For classification, a fully connected neural network is used as depicted in Figure 3. The classifier network consists of three fully connected (FC) layers with neurons 1024, 512, and 341, respectively. Each FC layer is followed by batch normalization (BN) and LeakyRelu. This deep neural network decides which trace needs SNR enhancement. In other words, the output is a binary decision. The decision is based on the SNR after MLSE, i.e., if SNR is greater than 40 dB then trace is assumed to be recovered on the other hand it is further processed using the deep neural network for SNR enhancement. The reason for selecting this threshold is that the trace with SNR =40 dB is considered as near-lossless [14]. The optimization parameters are the same as before except for $\lambda =10^{-4}$ and lr is kept constant.

### 3.4. Overall Work Flow

**Figure 4:**
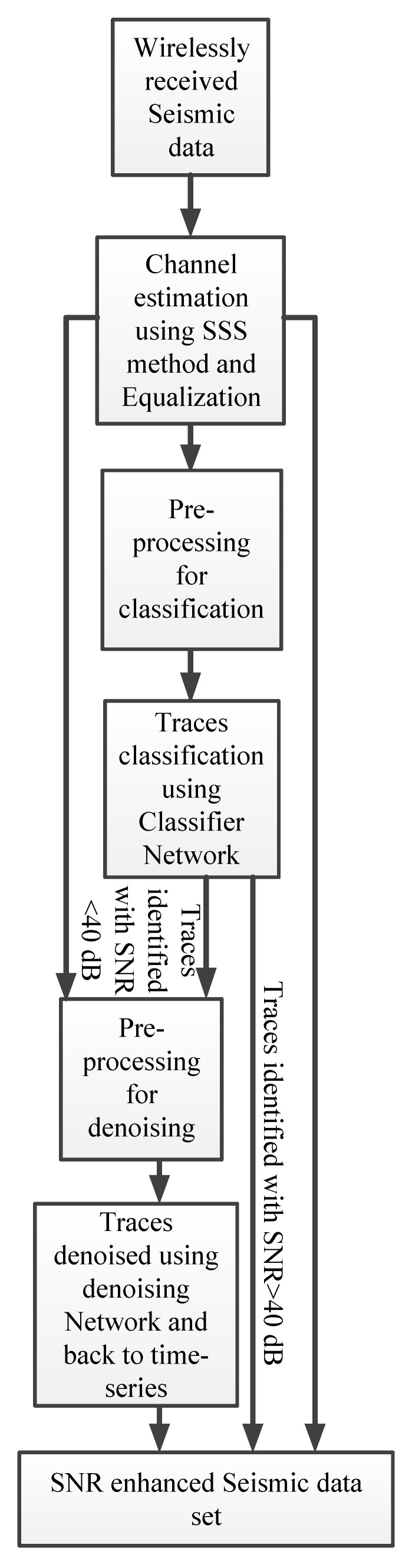
Flow chart for SNR enhancement of seismic data set.

Figure 4 depicts the overall workflow for the SNR enhancement. First, the received seismic data obtained through a wireless link are equalized using the SSS method and MLSE. Then, the data are classified and traces that required SNR enhancement are fed to the denoising network to obtain the denoised traces. For the reader’s convenience, the proposed method is summarized in Table 1.

## 4. Results and Discussion

To train the deep neural networks, synthetic data are generated using the Marmousi Model [15]. This model is often used in exploration seismology as a standard case study. The receivers are placed at a distance of 50 m in the horizontal axis and the shots are sequentially generated at the same location as every other receiver. The shot records are generated using the various seismic signatures, i.e., Ormsby wavelet with frequencies 10−15−65−70, 0−15−70−80, 0−5−45−70, 0−5−55−60, 5−10−55−60, 0−565−70, 0−5−45−50, and 10−15−50−55 Hz. The use of different frequencies for the Ormsby wavelet is to ensure well-trained deep neural networks that can be used for a variety of data sets. The frequency spectrum of the Ormsby wavelet is of trapezoidal shape which gives more flexible control on frequency domain than Ricker wavelet. This helps reconstruct the seismograms similar to the real seismic data. For the generation of synthetic data, the Matlab package [16] is used. The sampling frequency is set to 4 kHz. Furthermore, the training data are randomized before being given to the neural networks and then shuffled after every epoch. The input (predictors *P*) and output (targets *T*) training data are z-normalized for both neural networks as follows:

(10) $$\begin{array}{c} \bar{P}=\frac{P-\mu_{p}}{\sigma_{p}},\bar{T}=\frac{T-\mu_{t}}{\sigma_{t}}, \end{array}$$

where $\mu_{p}$($\mu_{t}$) and $\sigma_{p}$($\sigma_{t}$) are the mean and variance of *P*(*T*), respectively. Furthermore, 10% of the data are used for validation. The neural networks are first trained on the synthetic data obtained using the Marmousi model. To obtain noisy synthetic data, the raw data are converted from analog to digital and then randomly flipped 10% of the bits corresponding to a trace. The noisy data are mixed with the original raw data for training the classifier neural network.

In order to verify the performance, the classifier neural network is tested on publicly available seismic data from Utah Tomography & Modeling/Mitigation Consortium (UTAM) [17]. In this dataset, each trace has 4000 samples with the sampling frequency of $f_{s}=4$ kHz. Before the preprocessing stage, the data are cleaned by removing bad traces due to geophones not operating properly. The channel length is set to L=5 and Rayleigh fading is assumed. Furthermore, m=32, M=5, and Binary Phase Shift Keying (BPSK) modulation is used in the simulations.

**Figure 5:**
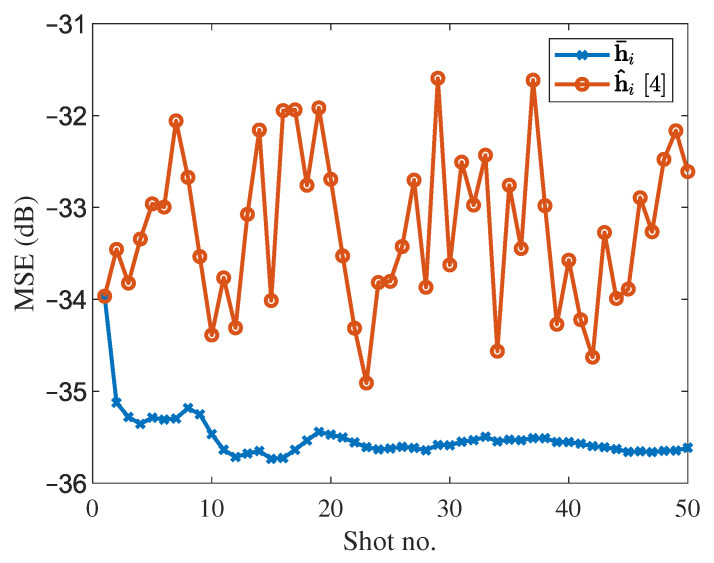
MSE comparison of SSS method and modified updated estimation at SNR =−5 dB.

Figure 5 shows a comparison of the state-of-the-art SSS method with the proposed one. The estimation of the channel impulse response is enhanced tremendously. As the estimation is leveling off around 50 shots, the final estimated channel impulse response is ${\bar{h}}_{50}$. Moreover, a large variation in estimation is observed for various traces in the case of the SSS method due to the random noise. Therefore, in some traces recovery might be worst and in turn, further seismic data processing steps like stacking will give very bad overall results (due to error accumulation).

**Figure 6:**
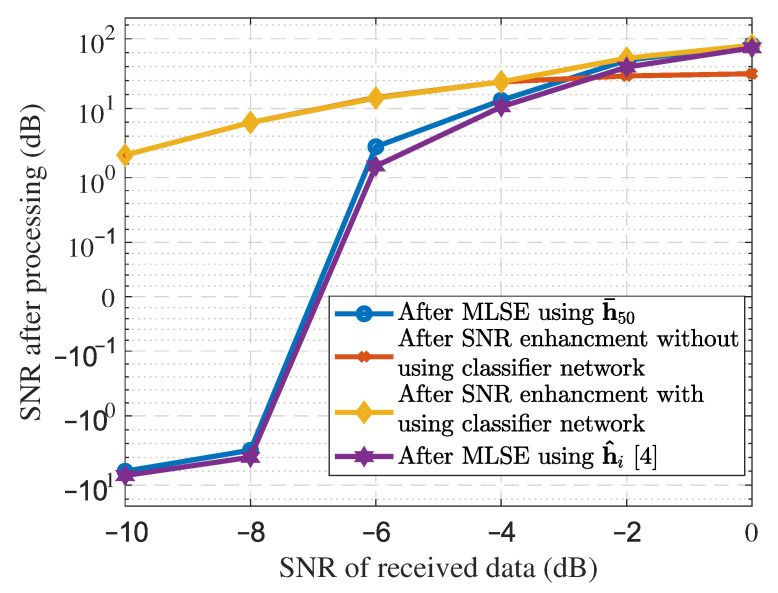
SNR enhancement using deep convolutional neural network, SNR of the received data y(n) versus SNR of the reconstructed traces after processing.

The SSS method, its proposed improvement, and SNR enhancement with or without the classifier network are compared and reported in Figure 6. SNR is obtained by averaging the respective values over all traces. It can be seen in Figure 6 that significant performance improvement is achieved through the use of pre-trained deep neural networks, in particular at high noise levels. For instance, at the SNR of −10 dB of the received data, a nearly 10 dB increase in SNR is achieved while at the SNR of 0 dB of the received data, about 120 dB increase in SNR is observed. It is noteworthy to argue that with only the SNR enhancement network the performance deteriorates at low noisy environments due to the robust estimation of the channel impulse response. The SNR of received data, in practical scenarios, is not known. Therefore, it is worth using a pre-trained classifier network without transmitting any training signal to the data center for estimating SNR. Furthermore, to the best of the authors’ knowledge, the proposed method is developed for the first time considering the wireless seismic system and, therefore, comparison with the other methods is not possible.

**Figure 7:**
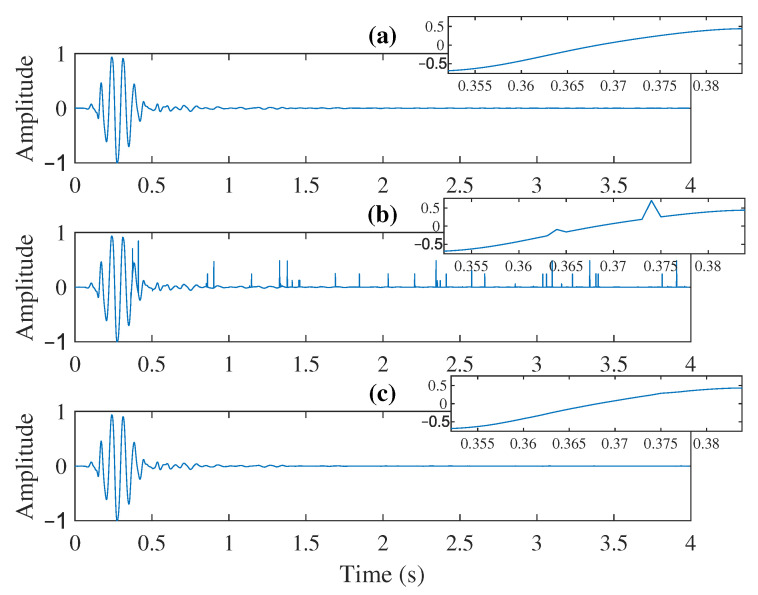
Single trace SNR enhancement (SNR of received data is −5 dB). (**a**) Original trace. (**b**) Reconstructed trace after MLSE. (**c**) Reconstructed trace after SNR enhancement. On the right side: zoomed view of panels (**a**–**c**).

In order to show the SNR improvement single trace, the proposed method is simulated for the SNR of received data of −5 dB. Figure 7 compares the reconstructed trace with the original trace. Figure 7a–c depicts the original trace, reconstructed trace after MLSE without denoising, and reconstructed trace after denoising, respectively. The improvement can be seen before and after using pre-trained neural networks. The spikes in the received data are pretty much removed by the denoising network which is clear from the zoomed version of Figure 7a–c. Some examples from the experimental results using real/field data with different levels of noise are shown in Appendix A.

**Figure 8:**
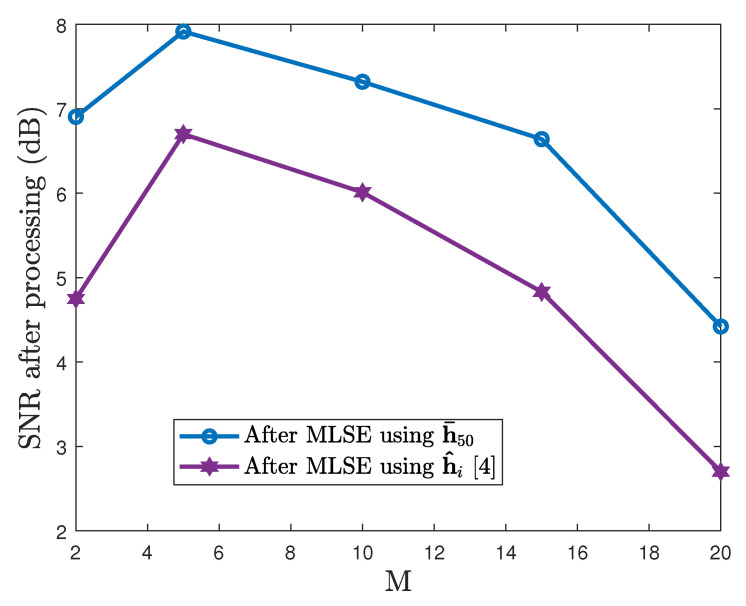
SNR enhancement versus various values of window size *M* (SNR of the received data is −5 dB).

The window size *M* is also crucial and the blind channel estimation performance depends on it. The blind algorithm highly is dependent on the structure of the channel matrix $H_{M}$. Therefore, it is shown in Figure 8 that when the window size *M* has a small value, the structure of the channel matrix is poor and the performance suffers. However, increasing the value of *M* beyond 15 (large channel matrix) causes the minimization error to increase, resulting in a decrease in performance.

## 5. Conclusions

Wireless seismic network poses major challenges given the gigantic amount of data that need to be transmitted from seismic sensors to the on-site data processing center. Most importantly, wireless transmission requires removing the wireless channel effects from seismic data. This is the most important factor that has been ignored in the previous studies for the realization of wireless seismic data transmission. While transmitting the seismic data wirelessly, impairments like interference, multi-path fading, and channel noise need to be considered. Therefore, in this work, a novel method is proposed for this purpose that comprises blind system identification and deep neural networks. The method works by training the deep neural networks offline and takes into account the stationary environment of the seismic data acquisition network. As a geophone already is responsible for transmitting a tremendous amount of the data under tight timing constraints, the proposed setup does not require sending any additional training signals to mitigate the channel effects. Furthermore, the proposed method is suitable for real-time implementation without the use of field data for the training of deep neural networks. Experiments show that the proposed method gives promising results when applied to the field data set.

## Figures and Tables

**Table 1:** Summary of the proposed method.

1. Seismic data with channel impairments.
2. Blind channel estimation using (3) and equalization using MLSE.
3. DFT of traces for classification.
4. Classification of traces in to two categories using classifier network.
5. STDCT of traces with SNR less than 40 dB for denoising.
6. Denoising of traces using denoising network.
7. SNR enhanced seismic data.

**Figure A1:**
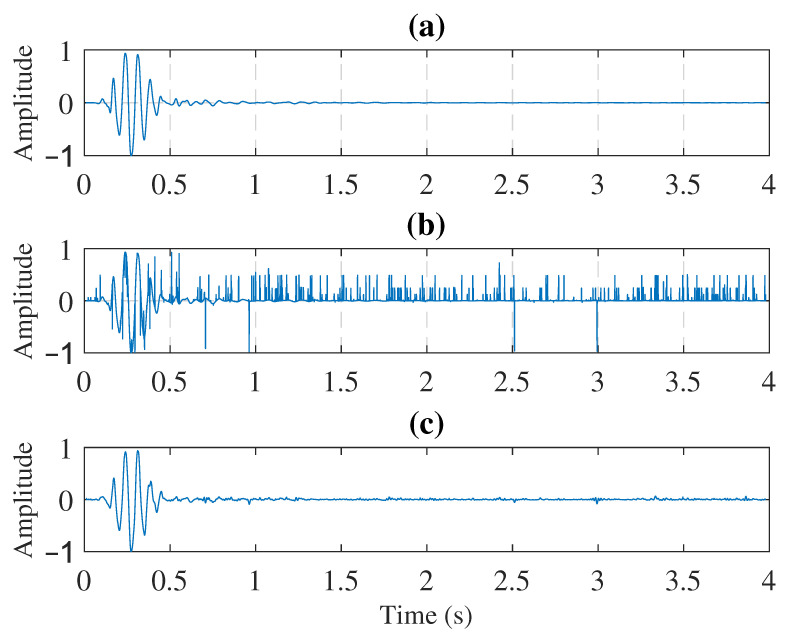
Single trace SNR enhancement (SNR of received data is −8 dB). (**a**) Original trace. (**b**) Reconstructed trace after MLSE. (**c**) Reconstructed trace after SNR enhancement.

**Figure A2:**
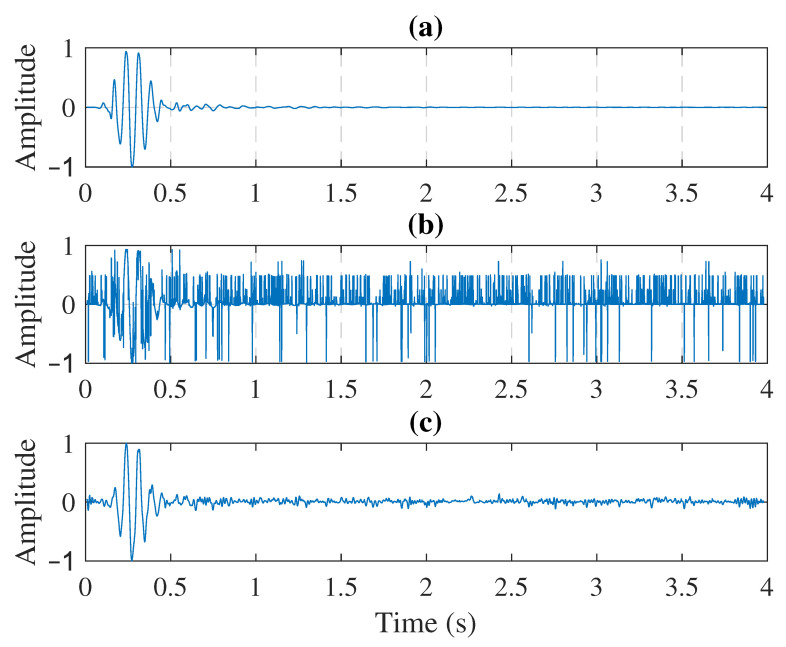
Single trace SNR enhancement (SNR of received data is −10 dB). (**a**) Original trace. (**b**) Reconstructed trace after MLSE. (**c**) Reconstructed trace after SNR enhancement.

## Data Availability

The data are available by contacting the corresponding author.

## Appendix A

The efficacy of the proposed method is further highlighted in Figure A1 and Figure A2. The proposed method efficiently suppresses the noise in the worst case when the SNR of the received data is −10 dB. Figure A1b and Figure A2b also confirm that the need for robust processing of the wirelessly transmitted seismic data is highly desirable.
